# Working together with people with intellectual disability to make a difference: a protocol for a mixed-method co-production study to address inequities in cervical screening participation

**DOI:** 10.3389/fpubh.2024.1360447

**Published:** 2024-05-23

**Authors:** Deborah Bateson, Jane Ussher, Iva Strnadová, Julie Loblinzk, Michael David, Ee-Lin Chang, Allison Carter, Sally Sweeney, Lauren Winkler, Rosalie Power, Caroline Basckin, Elizabeth Kennedy, Heather Jolly

**Affiliations:** ^1^The Daffodil Centre, University of Sydney, a joint venture with Cancer Council NSW, Sydney, NSW, Australia; ^2^Translational Health Research Institute, Western Sydney University, Sydney, NSW, Australia; ^3^University of New South Wales, Faculty of Arts Design and Architecture, School of Education, Sydney, NSW, Australia; ^4^University of New South Wales, Disability Innovation Institute, Sydney, NSW, Australia; ^5^Self Advocacy Sydney, Sydney, NSW, Australia; ^6^School of Medicine and Dentistry, Griffith University, Gold Coast, QLD, Australia; ^7^Family Planning Australia, Sydney, NSW, Australia; ^8^Kirby Institute, UNSW Sydney, Sydney, NSW, Australia; ^9^Australian Human Rights Institute, UNSW Sydney, Sydney, NSW, Australia; ^10^Faculty of Health Sciences, Simon Fraser University, Burnaby, BC, Canada

**Keywords:** inequity, intellectual disability, cervical screening, co-production, trauma informed care, self-collection HPV test, accessible information

## Abstract

**Introduction:**

Cervical cancer is one of the most preventable cancers yet remains a disease of inequity for people with intellectual disability, in part due to low screening rates. The ScreenEQUAL project will use an integrated knowledge translation (iKT) model to co-produce and evaluate accessible cervical screening resources with and for this group.

**Methods:**

Stage 1 will qualitatively explore facilitators and barriers to screening participation for people with intellectual disability, families and support people, healthcare providers and disability sector stakeholders (*n* ≈ 20 in each group). An accessible multimodal screening resource, accompanying supporting materials for families and support people, and trauma-informed healthcare provider training materials will then be co-produced through a series of workshops. Stage 2 will recruit people with intellectual disability aged 25 to 74 who are due or overdue for screening into a single-arm trial (*n* = 48). Trained support people will provide them with the co-produced resource in accessible workshops (intervention) and support them in completing pre-post questions to assess informed decision-making. A subset will participate in qualitative post-intervention interviews including optional body-mapping (*n* ≈ 20). Screening uptake in the 9-months following the intervention will be measured through data linkage. Family members and support people (*n* = 48) and healthcare providers (*n* = 433) will be recruited into single-arm sub-studies. Over a 4-month period they will, respectively, receive the accompanying supporting materials, and the trauma-informed training materials. Both groups will complete pre-post online surveys. A subset of each group (*n* ≈ 20) will be invited to participate in post-intervention semi-structured interviews.

**Outcomes and analysis:**

Our primary outcome is a change in informed decision-making by people with intellectual disability across the domains of knowledge, attitudes, and screening intention. Secondary outcomes include: (i) uptake of screening in the 9-months following the intervention workshops, (ii) changes in health literacy, attitudes and self-efficacy of family members and support people, and (iii) changes in knowledge, attitudes, self-efficacy and preparedness of screening providers. Each participant group will evaluate acceptability, feasibility and usability of the resources.

**Discussion:**

If found to be effective and acceptable, the co-produced cervical screening resources and training materials will be made freely available through the ScreenEQUAL website to support national, and potentially international, scale-up.

## 1 Introduction

In 2020 the Director General of WHO, Dr. Tedros Ghebreyesus, launched the Global Strategy for the Elimination of Cervical Cancer as a public health problem within the next hundred years (1). This previously unthinkable goal is achievable because of knowledge advances about the role of human papillomavirus (HPV) as the cause of almost all cervical cancers, and subsequent evolution of preventive technologies. Despite these advances, cervical cancer remains a disease of inequity, reflecting structural and psychosocial barriers to preventive care across the three pillars of HPV vaccination, cervical screening, and treatment of precancers (2, 3).

In Australia, cervical screening is provided through the National Cervical Screening Program (NCSP) with support from the National Cancer Screening Register (NCSR), which sends invitations and screening reminders to eligible people (4). In 2017 the program switched from two-yearly Pap tests from 18 to 69 years of age to five-yearly HPV tests from the 25 to 74 years (5). In 2022 a further policy change saw the introduction of universal access to self-collection of a vaginal sample without the use of a speculum, with the explicit aim of reducing screening barriers and increasing participation of under-screened people (6). As a result of these screening initiatives and the school-based HPV vaccination program, Australia is on track to eliminate cervical cancer by 2035 (7). However, elimination must be achieved equitably.

Compared with the general population, people with intellectual disability experience additional barriers to cervical screening due to stigma and discrimination, community misassumptions and non-inclusive healthcare practices (8–11). Lack of knowledge and awareness about screening and its importance in preventing cervical cancer among people with intellectual disability can result from a lack of accessible health promotion information (10, 12, 13) and the withholding of comprehensive sex education in schools, due to misplaced family and community-based fears that it may promote sexual activity or increase vulnerability to sexual abuse (14). Fears and anxiety about screening processes among people with intellectual disability can be compounded by poor clinical interactions with healthcare providers, who may lack skills and confidence to communicate effectively and to provide culturally safe screening services (15–18). De-prioritisation of screening due to competing health demands (16, 18, 19), as well as misassumptions about sexual activity and awareness of the need for screening on the part of family members, support workers and healthcare providers, are also well documented (16, 20). As pointed out by the Royal Commission into Violence, Abuse, Neglect and Exploitation of People with Disability (2023), 72% of women with intellectual disability have experienced violence since age 15 years, and 45% of women with intellectual disability have been sexually assaulted (compared with 29% of women with any type of disability) (21). These high rates of sexual trauma and assault, including child sexual abuse, experienced by people with intellectual disability compared with the general population (22), not only present an additional screening barrier, especially concerning an intimate speculum examination, but also may be overlooked as a factor determining need for screening (23).

In Australia, almost three-quarters of people who develop cervical cancer are under-screened or never-screened (24). To address screening inequities, the collection of cervical screening data through national registries to identify under-screened groups is critical. A recent national Swedish data linkage study reported very low rates of screening participation in women with intellectual disability compared with those without intellectual disability [OR 0.34 (95% CI 0.33 to 0.36)] (25). However, for most countries comprehensive data are lacking, including in Australia. The most recent peer-reviewed Australian data is from a 2010 general practice-based trial which documented screening rates as low as 10% among people with intellectual disability (26), compared to 57% in the general population at that time (27).

There is a dearth of published interventions to increase cervical screening for people with intellectual disability, and the few that exist generally have well-documented limitations including small sample sizes and limited statistical power (12, 13, 26). A randomised control trial (RCT) in the United States found that women with intellectual disability who received an educational intervention about cervical and breast screening (Women Be Healthy) did not have significantly higher rates of knowledge gain, compared with control participants (12), and subsequent modifications to the intervention yielded only a modest increase in knowledge (13). By contrast, a 2007 Australian RCT assessing the impact of a Comprehensive Health Assessment Program (CHAP) to improve interactions between adults with intellectual disability, their family member and general practitioner (GP) reported an eight-fold increase in Pap tests (95% CI 1.8–35) compared with the control group (28). However, as noted by the authors, this approach was unable to measure empowerment of family members and people with intellectual disability (26, 28).

This paper outlines the protocol of ScreenEQUAL, a co-designed two-stage mixed-methods study, which will be carried out over 3 years by a multi-disciplinary team comprised of expert disability researchers including a chief investigator with intellectual disability, clinician researchers and health promotion experts. It will complement the CHAP which in 2023, after further modifications, was implemented nationally through a joint Department of Health and Ageing and National Disability Insurance Scheme (NDIS) initiative (29). The ScreenEQUAL program of work, like the CHAP, aligns with the Australian government’s 2021–2023 National Roadmap for Improving the Health of People with Intellectual Disability (30), which aims to address serious health inequities faced by this group.

The primary aim of ScreenEQUAL is to evaluate the effectiveness of a co-produced accessible multimodal cervical screening information resource in supporting informed decision-making about screening by people with intellectual disability. In this study, ‘informed decision-making’ refers to meeting three criteria. First, the person is given information that makes sense to them. Second, the decision is not influenced or forced. Third, the person must have capacity to make the decision (31, 32). To ensure valid consent, this process must be adjusted to fit the person’s needs so they can understand and express themselves well. Capacity refers to being able to agree to a medical treatment knowing what it involves (33). The Mental Capacity Act outlines that “a person must be assumed to have capacity unless it is established that he lacks capacity,” and that incapacity can only be established if “all practicable steps” to support capacity have been attempted without success (34).

The secondary aims are to assess:

- uptake of cervical screening by participants with intellectual disability in the nine-months following the trial measured through data linkage;
- changes in health literacy, attitudes and self-efficacy of family members and support people in supporting cervical screening participation by people with intellectual disability;
- changes in knowledge, attitudes, self-efficacy and preparedness of healthcare providers in supporting and providing cervical screening for people with intellectual disability.

The objectives of Stage 1 of the study are to build on existing Family Planning Australia resources by (35):

- qualitatively exploring cervical screening facilitators and barriers for people with intellectual disability as perceived by people with intellectual disability themselves, their family members and support people, healthcare providers and disability sector stakeholders;
- co-producing an accessible information resource to support cervical screening, including the option of self-collection, for people with intellectual disability;
- co-producing accompanying supporting materials for families and support people to use when sharing the accessible screening resource with people with intellectual disability;
- co-producing trauma-informed training materials for healthcare providers.

The Stage 2 objectives are to evaluate the co-produced study outputs (b,c,d) through a single-arm main trial with participants with intellectual disability, and single-arm sub-studies with family members and support people, and healthcare providers.

**Figure 1:**
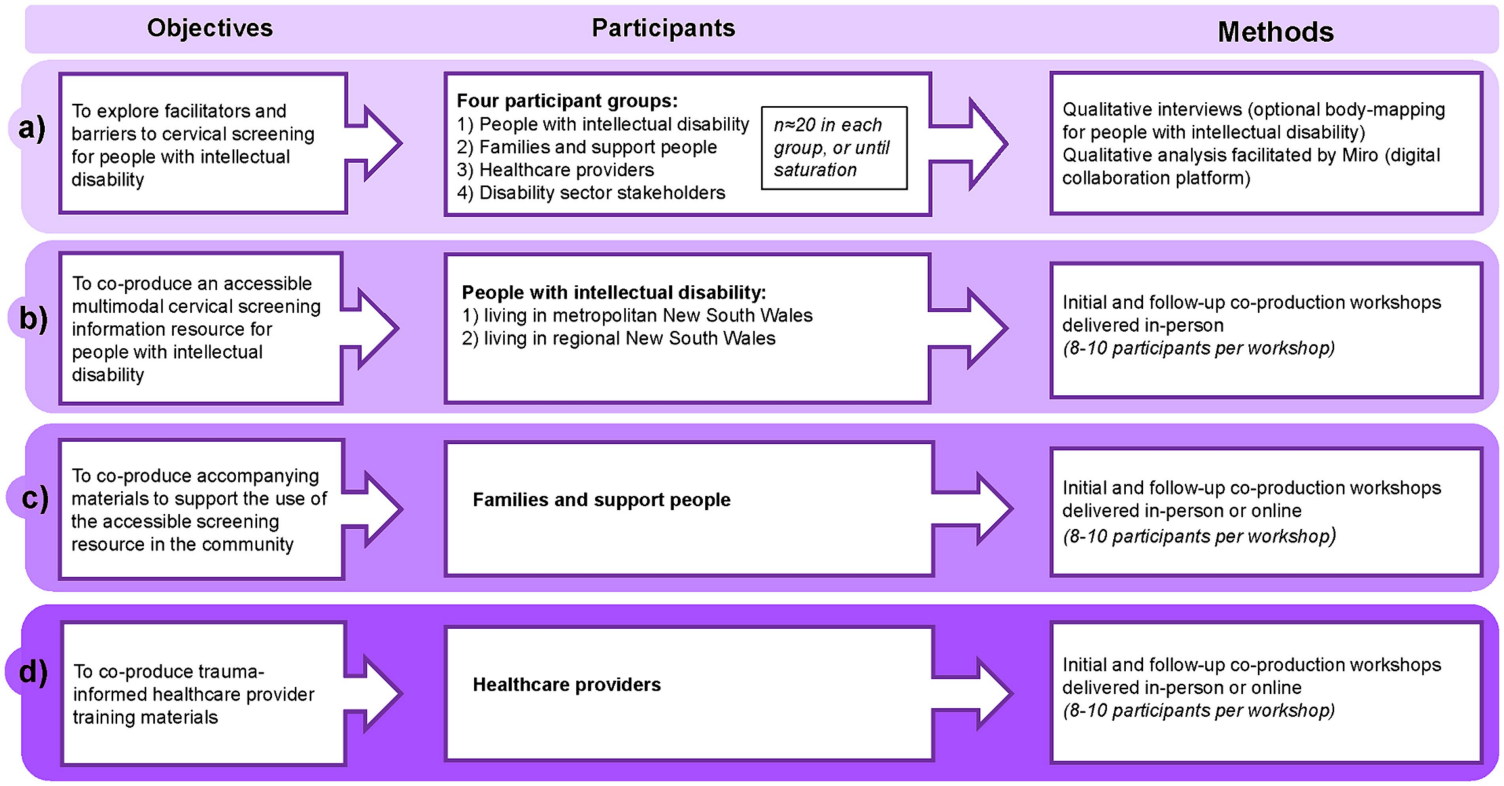
ScreenEQUAL Stage 1 overview.

**Figure 2:**
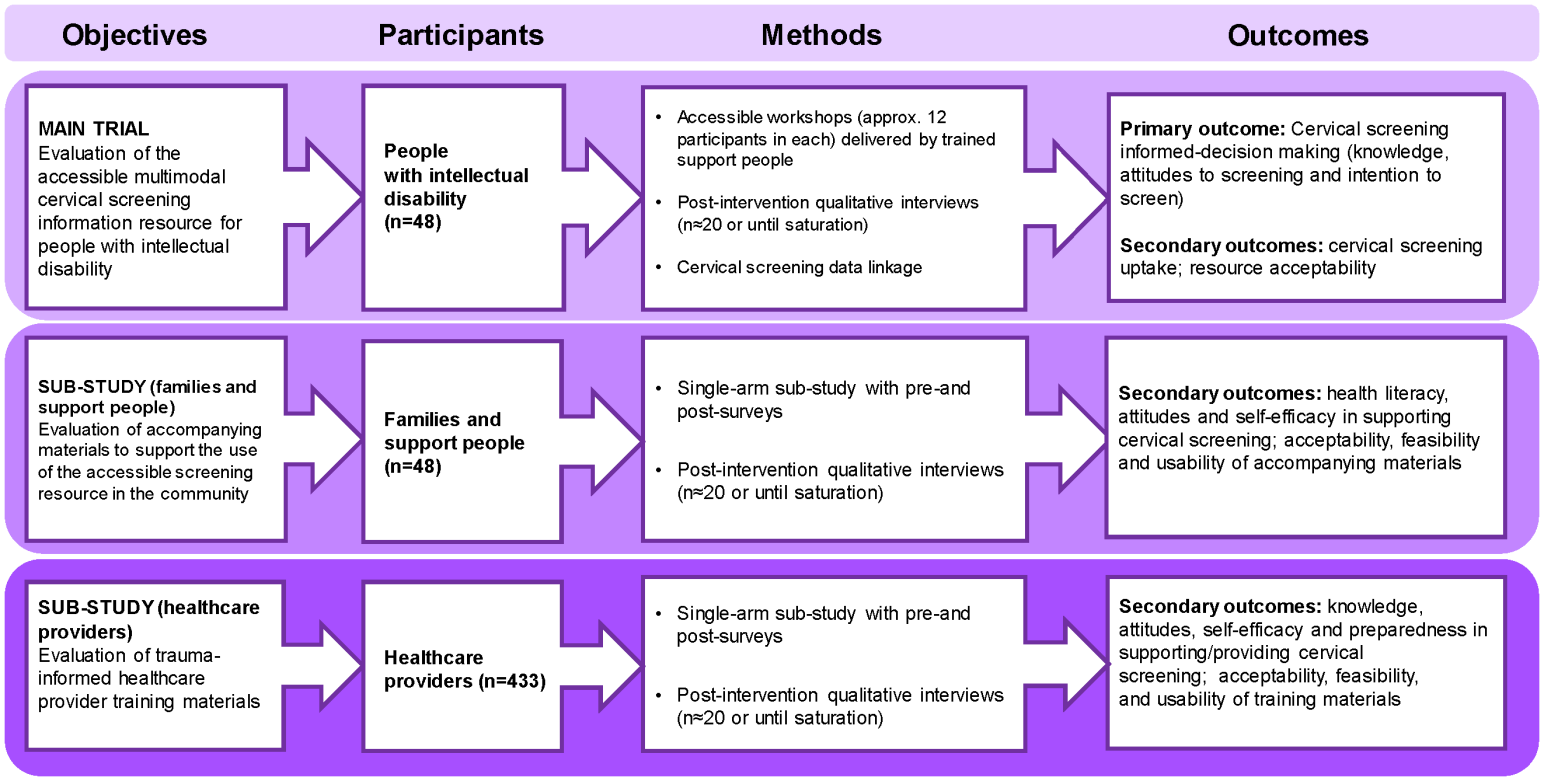
ScreenEQUAL Stage 2 overview.

**Figure 3:**
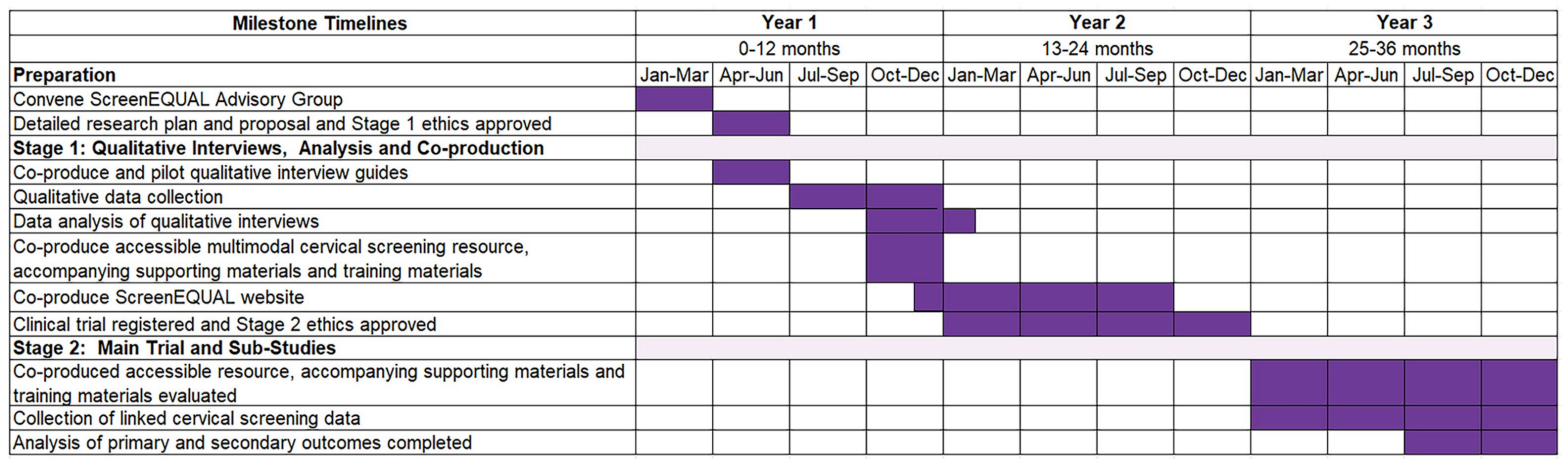
ScreenEQUAL study timeline.

Figures 1, 2 provide an overview of Stages 1 and 2 of the study, and the study timeline is shown in Figure 3.

## 2 Methods and analysis

### 2.1 Inclusive study methods

#### 2.1.1 ScreenEQUAL co-production study framework

The study will adhere to the principles of the inclusive research framework “Doing Research Inclusively: Guidelines for Co-Producing Research with People with Disability” (36), and an integrated knowledge translation (iKT) model of research co-production, whereby researchers’ partner with knowledge users throughout the research process to support translation of research findings into practice and policy (37).

It will also adopt a trauma-informed approach to minimise the risk of re-trauma of participants with intellectual disability due to the sensitive nature of cervical screening and the potential impact of a history of sexual assault or previous negative screening experiences (38). Trauma-informed practice is grounded in the understanding that trauma exposure can impact an individual’s neurological, biological, psychological and social development (38, 39), and seeks to address the barriers that people affected by trauma can experience when accessing health and care services.

A ScreenEQUAL Advisory Group will be convened, including people with intellectual disability and other stakeholders from the disability, cancer, and healthcare sectors, with representation across regional and rural, Aboriginal and Torres Strait Islander, culturally and linguistically diverse (CALD) and LGBTQ+ communities. Its governance structure will foreground members with intellectual disability representing grassroot community-based organisations. Two Advisory Group meetings will be held annually, with individual pre, and post meeting preparation and debriefing for members with intellectual disability, to optimise participation and mitigate risks of re-traumatisation. Grassroot organisations run by and for people with intellectual disability, who will be represented on Advisory Group, will be compensated for their time in line with community standards.

**Figure 4:**
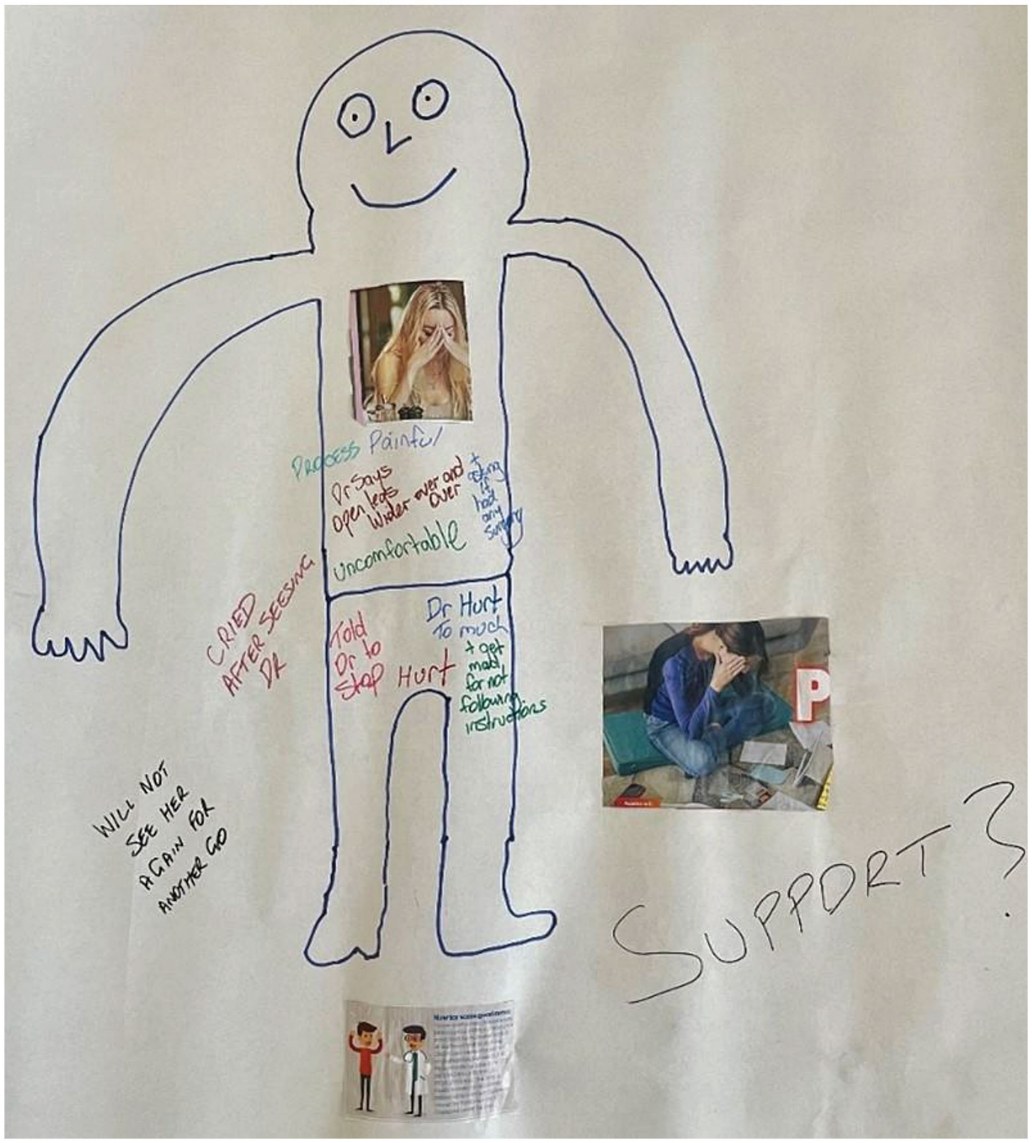
A de-identified body-map from the preparatory study phase.

Each research team member will undertake Easy Read training which will be universally used to ensure accessibility of study documents and communications (40). Stages 1 and 2 will include optional body-mapping, an arts-based participatory research method, for participants with intellectual disability, to align with participants’ preferences. Body-mapping involves the participant (or, if preferred, an expert disability researcher) tracing around their body to create a life-sized outline and being invited to fill their body outline by drawing or attaching pictures, writing words, or using colours, associated with their own experiences with cervical screening (41) (a de-identified example from the preparatory phase of the study is shown in Figure 4).

To foreground perspectives of people with intellectual disability in data analysis and interpretation, we will use creative, accessible, and participatory processes such as visual representations of the data, participatory videos, role-playing and/or co-analysis workshops, guided by the Disability Inclusive Guidelines (36) and recommendations from the study Advisory Group.

#### 2.1.2 Supporting a culturally safe trial design

The study was originally conceived as a cluster randomised control trial (cRCT) with clusters comprised of single healthcare providers and multiples of people with intellectual disability for whom they provide services, their family members and/or support people. However, a decision to shift to a pragmatic single-arm trial design for people with intellectual disability and sub-studies for independent groups of family members and support people, and healthcare providers, was made at the study planning stage. This shift was to ensure feasibility and an ethically sound approach after significant study design challenges became evident during community consultations. The extent of overall negative healthcare experiences exposed by the Royal Commission into Violence, Abuse, Neglect and Exploitation of People with Disability (21) published at the time of planning, helped inform the decision to use a single-arm trial design rather than clustering participants with intellectual disability together with their healthcare provider, family member and/or support worker, as was proposed in the original design. Other challenges with the initial design included the potential for distress for those allocated to a control group, the practicalities of recruiting sufficient participants with intellectual disability to meet the RCT sample size requirements (*n* = 200), and recognition that high rates of sexual assault amongst this population would necessitate additional supports beyond the research team’s capacity.

#### 2.1.3 Recruitment and gaining informed consent for participants with intellectual disability

The research team will collaborate with disability services and community-based advocacy organisations including those with which the research term has existing professional relationships to identify potentially eligible participants for study Stages 1 and 2. These include at least three grassroot self-advocacy organisations run by and for people with intellectual disability, and a large national private disability service supporting people with intellectual disability. The research team will also give presentations about the study at community disability forums to establish new connections with a diverse range of services and grassroot organisations.

The research team will consult the Advisory Group throughout the study to identify new sources of recruitment for potential participants with intellectual disability with diverse needs and backgrounds. This may include, if appropriate and where trusted relationships have been established, closed social media groups for people with intellectual disability, their families and support people as well as smaller grassroot community organisations and disability services.

**Figure 5:**
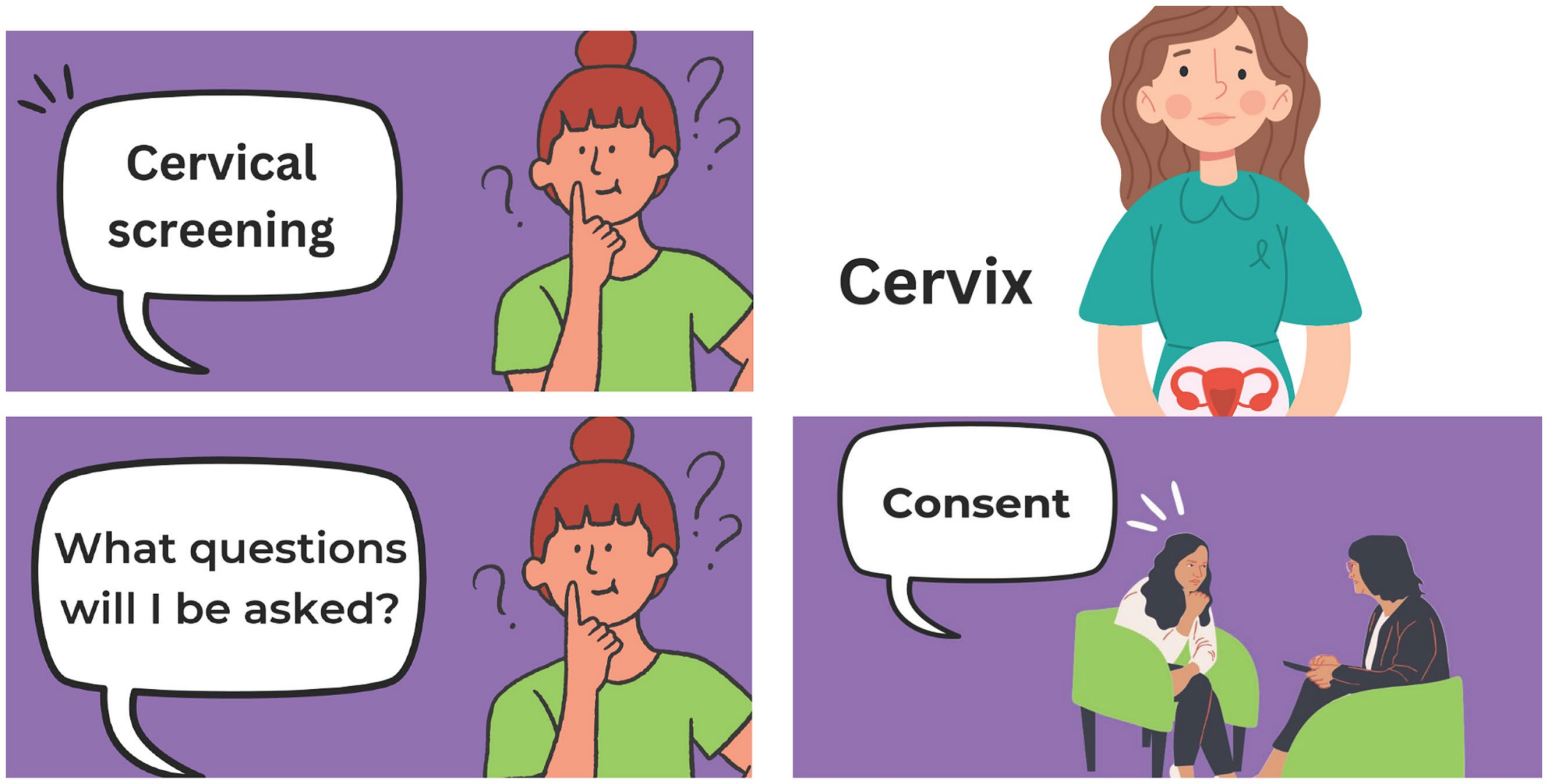
Screenshots of the of the accessible information video.

**Figure 6:**
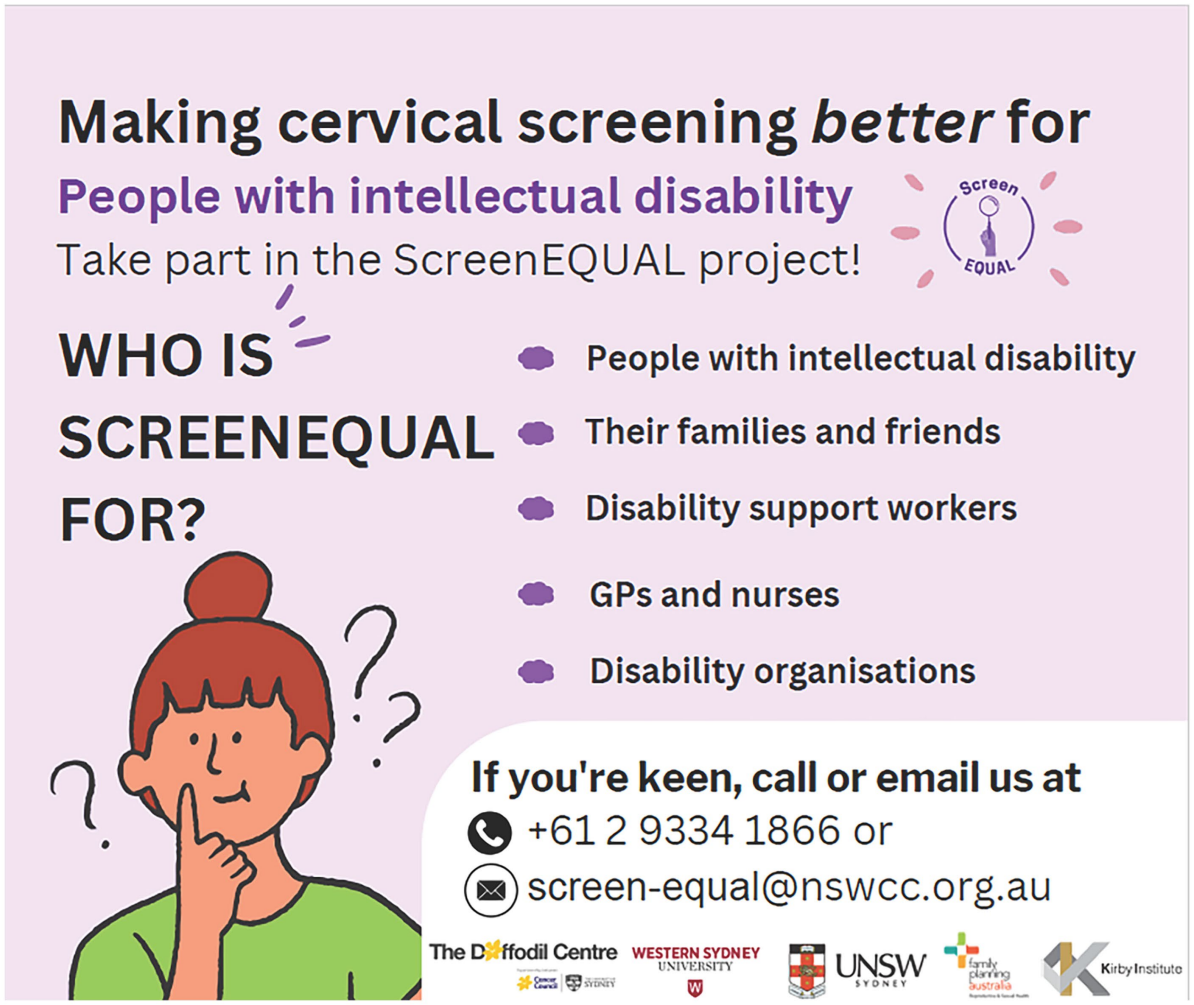
Screenshot of the easy read social media advertisement.

A member of the research team known to the disability organisation or service will be responsible for sending a letter of invitation requesting support for the study. Those interested will be invited to introduce the project to potentially eligible participants using a range of tailored approaches. Grassroot self-advocacy organisations will be invited to share an Easy Read participant information statement and study consent form (PISCF) with potentially eligible participants by advertising the study through their accessible websites and/or newsletters. Private disability services providing supported or independent living accommodation will be invited to share information about the study via their website and/or online newsletters, and to provide copies of the Easy Read PISCF to relevant site managers, who can in turn provide in-person information and the PISCF to eligible clients. The PISCF also includes links to an accessible information video (Figure 5) (40). This approach aims to make the study aims easily understood and to facilitate voluntary informed consent by participants. Potential participants can indicate their interest by contacting the researchers directly by telephone and/or email. Recruitment will also be supported by posting Easy Read social media advertisements (e.g., X – formerly Twitter, LinkedIn, Facebook) (Figure 6), and ‘snowballing’ whereby people with intellectual disability recommend participation to their friends, which is more likely if they have had a positive experience with the study.

The initial consent processes for study participation and all qualitative interviews (including participation in workshops) with participants with intellectual disability will be conducted by the expert disability researchers in the field of applied research in intellectual disability, including the chief investigator with intellectual disability. Mitigation strategies will be in place for participants who show signs of distress. This may include cessation of an interview, and follow-up with the participant and their support person to check whether additional support is needed through referral to a regular general practitioner (GP) or an appropriate counselling service. Participants in other groups who experience distress will also be provided with support through appropriate services, if required.

During the consent process, participants will have the opportunity to meet the interviewers at a location of their choice and ask any questions, prior to their interview. This process is important in enabling participants to develop rapport with the researchers conducting the interview, before deciding whether to take part in the study. The interviewers will explain the study in an accessible way using the Easy Read PISCF. The chief investigators conducting the interviews with people with intellectual disability are experienced in using alternative methods of communication, such as augmentative and alternative communication (e.g., pictograms, electronic speech generating devices) or arts-based methods (e.g., photographs, body-mapping) to engage potential participants with high support needs, including those with complex communication needs (42). A continuous (written and verbal) consent process will be used before, during, and after data collection. This will include checking with the participant that they continue to consent to their data being collected and analysed after their participatory interview, or whether they would like to withdraw from the study without penalty. Accessible information about the study, and the co-produced cervical screening information resource, supporting materials and training materials will be available on a co-produced ScreenEQUAL website.

### 2.2 Study setting

Participants with intellectual disability will be recruited from metropolitan, regional and rural areas of New South Wales, Australia, due to the necessity of in-person contact with the research team. Participants in the other groups (families, support people, healthcare providers and disability sector stakeholders) will be recruited from across Australia.

### 2.3 Stage 1 methods and analysis

Semi-structured interviews will be conducted to explore facilitators and barriers to cervical screening experienced by people with intellectual disability. A series of workshops will then be held to co-produce a suite of resources and materials to support cervical screening informed decision-making by people with intellectual disability (Figure 1).

#### 2.3.1 Stage 1 participants

The eligibility criteria for participants in Stage 1 of the study is shown in Table 1. Recruitment of people with intellectual disability will adhere to the inclusive approaches in section 2.1.3. Family members and support people, healthcare providers and disability sector stakeholders will be identified through the researchers’ professional networks and the Advisory Group. Potential participants will be emailed a link to an accessible project summary and a secure form to provide their name and telephone number. Healthcare providers will be able to opt to continue to the online consent form with an email alert sent to the research team signalling a new participant. Participants will be remunerated for their time in line with community standards.

**Table 1:** Eligibility criteria for Stage 1 qualitative interview participants.

Participant group	Eligibility criteria
People with intellectual disability	Eligible for screening in the NCSP (have a cervix and are aged 25 to 74 years); live in NSW; can communicate their experiences in English using either verbal or non-verbal communication (with augmentative and alternative communication tools if needed).
Family members and support people	Family member or support person (unpaid or paid, for example, disability support worker) of a person/people with intellectual disability who are eligible for screening in the NCSP; live or work in Australia; speak English.
Healthcare providers	GP or nurse working with people with intellectual disability who are eligible for cervical screening in the NCSP; practice in Australia; speak English.
Disability sector stakeholders	Senior leader in Australian disability sector organisation or government agency with an interest in provision of healthcare for people with intellectual disability; speak English.

#### 2.3.2 Qualitative semi-structured interviews and body-mapping

Semi-structured interviews with participants with intellectual disability will be co-conducted in-person at a disability organisation or other venue of their choice, or online via Zoom (Version 5.0.2), and will take approximately one hour. Participants will be offered the option of an additional in-person body-mapping session which will take approximately 2 to 3 h. Semi-structured interviews with family members and support people, healthcare providers and disability sector stakeholders will be conducted online via Teams or Zoom, by telephone, or in-person, depending on their preferences and will take 30 min to one hour, depending on the breadth of information provided by the participant. The interview domains and accompanying guides for each participant group will be based on existing published studies where feasible and developed through an iterative process with feedback from our multi-disciplinary team members and Advisory Group (Table 2 provides an overview of the domains).

**Table 2:** Overview of the Stage 1 qualitative interview domains for each participant group.

Interview domains	Participants			
	People with intellectual disability	Families and support people	Healthcare providers	Disability sector stakeholders
Demographics	x	x	x	x
Professional background			x	x
Knowledge of cervical screening	x	x		
Experiences of cervical screening	x			
Supporting or delivering cervical screening		x	x	x
Facilitators and barriers to cervical screening	x	x	x	x
Perceived role of self-collection for people with intellectual disability	x	x	x	x
Unmet information and training needs	x	x	x	x

Interviews will be audio-recorded and uploaded onto a secure online University of Sydney system using the participant’s unique study pseudonym and study identification number. For participants with intellectual disability, audio-files will be transcribed verbatim, using a combination of a transcriber and Happy Scribe software (43). The names of places and people identified in the interview, such as a local GP practice or healthcare provider, will not be transcribed and referred to only in general terms to further protect the anonymity of the participant. Integrity checking will be carried out for each transcription by the research team.

##### 2.3.2.1 Qualitative data analysis

The sample sizes for the semi-structured interviews will be informed by information power (44). A high level of information power will be identified when a highly rigorous process of analysis has been applied (analytical sufficiency) and the richness of the information generated is sufficient to answer the study aims (data sufficiency). The appraisal of information power will include a detailed review of the first three interviews delivered within each of the four participant groups (*n* = 12 interviews) by the chief investigators with extensive qualitative expertise. The expert chief investigators will then provide detailed feedback to the research team about whether the interviews are highly relevant for the research question and add new knowledge to the field. They will continuously review the quality of the dialogues, or at least monthly, before closing data collection. Reflexive thematic analyses of the interview responses will include a process of familiarisation with the transcripts, generation of codes and coding trees and the construction of overarching themes, followed by review and categorisation of key themes (45, 46). Reflexivity will be facilitated through ongoing group discussion, listening exercises and through team members being invited to take part in a body-mapping exercise, to understand the experience of participants. We will use NVivo (Version 14) software to facilitate coding of participants’ responses. To support accessibility, discussion of themes will be facilitated by Miro, a digital collaboration platform.^1^

#### 2.3.3 Co-production workshops

Stage 1 qualitative interview findings will be used to inform a series of initial and follow-up co-production workshops. All participants will be remunerated in line with community standards.

- **Co-production of an accessible cervical screening resource for people with intellectual disability**
  The resource for people with intellectual disability will be multimodal, incorporating Easy Read information and video materials to support accessibility for people with high support needs. It will build on the Family Planning Australia *Just Checking* resource series (47) which supports cancer screening for people with intellectual disability, their families and support people. Initial and follow-up co-production workshops (one of each in a metropolitan and a regional setting) with approximately 8 to 10 people with intellectual disability, each lasting approximately 4 h, will be facilitated by Family Planning Australia team members and the expert disability researchers. The initial workshops will set the scene about why cervical screening is important and explore how the existing resources could best be adapted to fit current community needs. Follow-up workshops with the same participants will gain feedback following resource modifications. The researchers will also collect qualitative data during the workshops through audio-recording of the group discussion. The data will be transcribed and analysed as described earlier.
- **Co-production of supporting materials for families and support people**
  Initial and follow-up co-production workshops with approximately 8 to 10 family members and support people will explore how these groups could use the accessible cervical screening resource in the community to support screening for people with intellectual disability. The initial workshop will explore how existing Family Planning Australia supporting materials could be adapted to meet the needs of this group. Modification of the materials will also be informed by the outcomes of the co-production workshops with people with intellectual disability, and feedback on the modified materials will be gathered at the follow-up workshop.
- **Co-production of supporting materials for healthcare providers**
  Trauma-informed healthcare provider training materials including extension of the existing Family Planning Australia *Supporting Decision-making Tool* (48) will developed by the clinician researchers, informed by the co-production workshop outcomes with people with intellectual disability and family members and support people. Initial and follow-up workshops will then be held with approximately 8 to 10 healthcare providers to explore how the training materials could be best used in clinical practice and determine how the training materials could be further modified to meet the needs of healthcare providers in supporting and delivering cervical screening for people with intellectual disability.

#### 2.3.4 Development of the ScreenEQUAL website

An accessible ScreenEQUAL study website will be developed alongside the co-produced materials, informed by the co-production workshops and with input from the Advisory Group. It will be used to house the study materials and news items relevant to the study.

### 2.4 Stage 2 methods and analysis – main trial and sub-studies

#### 2.4.1 Main trial participants – people with intellectual disability

People with intellectual disability will be eligible for participation in the single-arm main trial if they are due or overdue for cervical screening in the NCSP (age 25 to 74 years with a cervix) and can communicate verbally or non-verbally including through augmentative and alternative communication (Figure 2). Participation in Stage 1 will preclude participation in Stage 2 to reduce the chance of trial participants having enhanced knowledge of cervical screening prior to the intervention (Table 3). A separate consent process will seek permission for the research team to access participants’ cervical screening histories from the Cancer Institute NSW Cancer Screening Register at a 9-month timepoint after the trial. Participants who do not consent to their screening history being accessed, will not be excluded from the trial. Participant recruitment and processes for gaining informed consent are outlined in Section 2.1.3.

**Table 3:** Eligibility criteria for Stage 2 main trial and single-arm sub-study participants.

Participant group	Eligibility criteria
People with intellectual disability	Person with intellectual disability; due or overdue for cervical screening in the NCSP (age 25 to 74 years with a cervix); can communicate verbally or non-verbally (including through augmentative and alternative communication); has not participated in Stage 1 of the study
Family members and support people	Family member or support person (unpaid or paid, for example, disability support worker) of a person/people with intellectual disability who are eligible for screening in the NCSP; live or work in Australia; speak English.
Healthcare providers	GP or nurse who works with people with intellectual disability who are eligible for screening in the NCSP; practice in Australia; speak English.

#### 2.4.2 Main trial design

The main trial is a single-arm study aimed at evaluating the primary study outcome (a change in informed decision-making about cervical screening by people with intellectual disability). An accessible informed decision-making tool will be created based on existing tools and surveys informed by the research team, Advisory Group members with intellectual disability and experts in health literacy. The modified tool will collect data via interviews using a brief set of pre-intervention open-ended accessible questions across three domains: knowledge, attitudes to screening and intention to screen (49–52). The trial will serve as a pilot study for a larger appropriately powered validation trial.

People with intellectual disability (*n* = 48) will be invited to join one of 5 or 6 accessible workshops of approximately 10 people with intellectual disability and 2 to 3 trained support people, facilitated by members of the research team. The workshops will last approximately 2 to 3 h. Support people will be identified through Family Planning Australia networks to participate in a pre-workshop session where they will receive training according to a structured session plan on how to use the co-produced resource with people with intellectual disability. The trained support people will then use the resource with workshop participants in a way that simulates what would happen in a community setting (the intervention). Individual participants with intellectual disability will be supported to complete a brief set of pre-intervention open-ended accessible questions to assess informed decision making at the start of the workshop which will be repeated at the end of the workshop or within the next week depending on the participant’s choice. Qualitative data regarding the overall perceptions of the co-produced resource will be gathered during the workshops by the research team through audio-recording of the group discussion.

A subset of participants with intellectual disability (*n* ≈ 20 or until saturation is reached) will be invited to participate in post-intervention qualitative interviews, with the option of additional body-mapping, to provide further insights into perceptions of the resources and to support the interpretation of the study outcomes (41).

#### 2.4.3 Main trial sample size calculations

The sample size calculation is based on a difference in the proportion of people with intellectual disability making an informed decision about cervical screening pre-and-post the intervention (53). Noting that making an informed decision may not necessarily lead to a person having a screening test due to barriers such as a history of sexual assault, a baseline proportion of 20% was selected based on the last published national Australian figure of 10% screening uptake for this group (26). It was estimated that 44 participants would be needed to detect a change in the proportion (%) of those assessed as being able to make an informed decision (across the domains of knowledge, attitudes, and intention to screen) from 20% pre-intervention to 50% post-intervention, with power and alpha set at 80 and 5%, respectively. Due to the novelty of our study in terms of participants and intervention, no known estimates were available for pre-post correlation between the items in the modified informed decision-making tool or outcome variability at both time points. Consequently, a conservative approach was adopted by assuming there was zero correlation between pre-post observations measured by the modified tool and that these observations displayed maximum variability, i.e., standard deviation = 0.5 (54). Assuming a dropout rate of 10%, the adjusted sample was increased to 48.

#### 2.4.4 Data linkage

To determine participants’ screening histories and participation in the NCSP in the subsequent 9-months after the trial, linked cervical screening data within the NCSR records, held by the Cancer Institute NSW, will be accessed, and managed according to mandated data security measures (55). These data will also allow determination of whether the most recent cervical screening test (if any) since the trial was self-collected or collected by a clinician.

#### 2.4.5 Sub-study participants and study design – families and support people

Family members and support people of people with intellectual disability will be recruited into a single-arm sub-study (*n* = 48; see Table 3 for eligibility criteria). Over a 4-month timeframe this group will receive the co-produced accessible cervical screening resource and accompanying supporting materials about how it can be used to support people with intellectual disability to undertake cervical screening (the intervention). These resources will be housed on the ScreenEQUAL website, and their use will be tracked by fit-for-purpose methods including Google Analytics. Participants will be invited to complete pre-post surveys delivered online, by mail, or in-person, depending on their preference. A subset of participants (*n* ≈ 20 or until saturation is reached) will be invited to join post-intervention qualitative interviews. The surveys and interview domains will be informed by existing surveys, Stage 1 outputs and the Advisory Group, and will be piloted by family members and support people prior to the trial.

#### 2.4.6 Sub-study participants and study design – healthcare providers

Healthcare providers, who offer services for people with intellectual disability will be recruited into a single-arm sub-study (*n* = 433; see Table 3 for eligibility criteria). Over a 4-month timeframe, this group will receive the online training materials aimed at supporting delivery of trauma-informed cervical screening care for people with intellectual disability. Training uptake and engagement will be tracked using fit-for-purpose approaches. Participants will be invited to complete online pre-post surveys and a subset of participants (*n* ≈ 20 or until saturation is reached) will be invited to participate in post-intervention qualitative interviews. The surveys and interview domains will be informed by an existing healthcare provider survey (56), Stage 1 findings, and the Advisory Group, and will be piloted by healthcare providers prior to the trial.

#### 2.4.7 Sub-study sample size considerations

Sub-study sample size calculations are based on a difference in the pre-post intervention survey outcomes (57). Given the lack of baseline data related to sub-study outcomes related to health literacy, attitudes and self-efficacy for families and support people, the sample size (*n* = 48) derived for the main trial was also considered to be adequate to detect a change of 30% in pre and post measures for this group. For healthcare providers, it was estimated that a sample size of 393 would be needed to detect a change in preparedness to provide cervical screening from 65% pre-intervention to 75% post-intervention based on a recent Australian cervical screening survey (56). As per the other sample size calculations, a conservative approach was undertaken. Assuming a dropout rate of 10%, recruitment of 433 healthcare providers would be required (54).

### 2.5 Outcome measures and data analysis

#### 2.5.1 Outcome measures

The study outcome measures align with the study aims. Triangulation of quantitative and qualitative outcome measures will be used to evaluate pre-post differences for the main trial and sub-studies. The qualitative outputs will provide insights into participants’ perceptions of the resources and facilitate the interpretation and explanation of the primary and secondary outcomes.

The primary outcome measure is informed decision-making about cervical screening by people with intellectual disability across the domains of knowledge, attitudes to screening and intention to screen, measured by an accessible modified tool (49–52). The modified tool will collect data during accessible workshops in the main trial as described in section 2.4.2. Given the lack of an existing validated tool to measure informed decision-making by people with intellectual disability about health screening, the research team led by the chief investigator with intellectual disability and health literacy experts, aims to modify an existing tool (49, 50). The tool will likely be employed using interviews to assess knowledge, attitudes and intention to screen including open-ended accessible questions around the role of screening in preventing cervical cancer, screening options, including the choice of a self-collected test, and the likely consequences of screening including recommended follow-up after an abnormal test result. Similarly, participants’ attitudes towards screening may include agreement or disagreement with accessible statements that cervical screening is ‘beneficial’ or ‘harmful,’ while intention to screen may be assessed through an accessible scale with responses ‘I will,’ ‘I am not sure,’ ‘I will not.’

Secondary outcome measures (assessed during the main trial sub-studies) are:

- participation in the NCSP by people with intellectual disability, including uptake of self-collection versus clinician collection;
- pre-post differences in family members’ and support peoples’ health literacy, attitudes, and self-efficacy in supporting cervical screening for people with intellectual disability;
- pre-post differences in healthcare providers’ knowledge, attitudes, self-efficacy and preparedness in supporting and providing cervical screening for people with intellectual disability.

The acceptability, feasibility and usability of the co-produced information resources and training materials for families and support people and healthcare providers will also be assessed through post-intervention surveys and the qualitative interviews, supplemented by data tracking of engagement by families and support people and healthcare providers with the resources and training materials on the ScreenEQUAL website. Final adjustments to the resources and training materials required as a result of the trial outcomes will be made at the conclusion of the study.

#### 2.5.2 Data analysis

Qualitative data collection and thematic analysis will follow the principles and processes described earlier. Descriptive statistics will summarise participants’ baseline characteristics in the main trial and sub-studies. The impact of the intervention (i.e., effects) on the primary and secondary outcomes will be assessed using multivariable logistic regression modelling. This approach adjusts for the data being correlated because of the pre-and-post study design. Effects will be reported as adjusted odds ratios (ORs) with 95% Confidence Intervals (CIs). Univariable logistic analyses will be used to identify potentially confounding variables such as age and place of residence. Two-sided *p*-values less than 0.05 will be considered as significant. Stata Version 18 (StataCorp LLC, College Station, TX) will be used to analyse data.

### 2.6 Dissemination of study findings

Study findings will be published in peer-reviewed journals and disseminated through the research team’s networks to key stakeholders including national and state and territory governments. If found to be effective and acceptable, the co-produced cervical screening resource, supporting materials and training materials will be made freely available at the study conclusion through the ScreenEQUAL website to support national, and potentially international, scale-up. Study findings will be also written about in an Easy Read report, available on ScreenEQUAL website and distributed to grassroot organisations across Australia.

### 2.7 Confidentiality and data storage

All participant data from Stages 1 and 2 will be kept confidential, except in situations of imminent risk to self or others or suspicion of child or elder abuse. No information from people with intellectual disability will be shared with family members, support workers, or participant’s healthcare providers except in situations required by law. Confidentiality will be maintained by allocating each participant a unique study identification number and study pseudonym, and by numerically coding all data and keeping all electronic data in a highly restricted University of Sydney computer drive, using password protected electronic documents. Hard copies of participant consent forms, and/or questionnaires will be scanned and saved electronically, and the original versions shredded.

Participants’ survey data will be completed in REDCap, a secure online survey, and data management system (58). Semi-structured interview data will be stored as MS Word documents on a secure SharePoint Network. Body-maps will be digitised, and all electronic data will be kept in an electronic password-protected file on a highly restricted university computer drive, accessible to authorised individuals (USYD Share Point) (59).

## 3 Discussion

Available data suggest that people with intellectual disability have disproportionately low rates of cervical screening compared with the general population, (60–63) yet there are few published studies of interventions to increase screening participation for this group. Barriers occur at multiple levels, including at societal, health service, healthcare provider, support worker, family, and individual levels (8–11). Stage 1 of this study seeks to address this gap, through in-depth exploration of facilitators and barriers to cervical screening, from the perspective of people with intellectual disability, their families and support people, healthcare providers, and disability sector stakeholders. Findings from Stage 1 will help inform co-production workshops to produce an accessible multimodal cervical screening resource which can be used by the intellectual disability community, as well as by families, support people, healthcare providers and disability organisations to support screening equity. Importantly, it will contain updated information to increase awareness of the option of self-collection of a cervical screening test, which has been universally available for anyone eligible for the Australian NCSP since mid-2022 (64). Self-collection can potentially overcome barriers related to a speculum examination (6), which may be heightened for this group due to high rates of sexual trauma and negative past screening experiences (21, 22). It is also essential that awareness is raised not just for people with intellectual disability but also for their families and support people who may have low levels of health literacy about recent screening advances.

This study will also support the co-production of training materials for healthcare providers who, as highlighted by the recent Royal Commission into Violence, Abuse, Neglect and Exploitation of People with Disability (21), can lack the skills, knowledge and confidence to provide trauma-informed care. The increased rates of sexual assault and abuse for women and girls with disability exposed by the Royal Commission (21), highlight the necessity of approaches to prevent triggering and re-traumatisation through cervical screening which could potentially lead to the re-experiencing of thoughts, feelings or sensations experienced at the time of such a traumatic event or circumstance in a person’s past (39). In addition to training in trauma-informed care, a recent systematic review shows that clinician training is central to addressing healthcare providers’ attitudes to and lack of education in informed consent for people with intellectual disability (32). Extension of the Family Planning Australia *Supporting Decision-making* Tool (48) as part of the co-produced healthcare provider training materials will contribute to filling this gap with implications for informed consent beyond cervical screening to other healthcare procedures. The systematic review also recommends co-production of accessible information resources and further inclusive research into informed consent for people with intellectual disability to make this process equitable and accessible (32). The ScreenEQUAL cervical screening resource for people with intellectual disability, together with supporting materials for families and support people and healthcare provider training materials, will help address these inequities and lay the foundations for an accessible informed consent process for cervical screening.

The strengths of this study lie in its comprehensive co-produced program of work with people with intellectual disability and the inclusion of families and support people as well as healthcare providers. While many resources aimed at supporting cancer screening for people with intellectual disability purport to be accessible, few appear to be truly co-produced. This is likely due to a lack of knowledge and understanding of co-production and perceptions of it being too difficult, preconceptions about patients’ limitations to coproduce, fear of change and power imbalances and financial and time constraints (65, 66). By contrast, ScreenEQUAL adheres to the principles within the Convention on the Rights of Persons with Disabilities of ‘nothing about us without us’ (67) and will support a holistic approach to empowering people with intellectual disability to make an informed decision to participate in screening, and to optimise their experience through trauma-informed care. We will also prioritise co-production and intervention evaluation workshops in regional areas, where access to health information resources and healthcare professionals is limited, to ensure community needs are met for the most marginalised populations. Another strength lies in the use of a modified accessible cancer screening informed decision-making tool, which will be developed within the context of the study and validated in the future for wider use in research evaluating the impact of different cancer screening interventions for people with intellectual disability.

Limitations reflect the potential lack of generalisability of the study population to the national population of people with intellectual disability. It is possible that participants with intellectual disability in the main trial may have greater awareness of cervical screening through shared networks with participants in Stage 1 of the study. Healthcare providers volunteering to participate in the study are also likely to have greater investment in providing cervical screening to people with intellectual disability, and may be more likely to be aware of, and to practice, trauma-informed care than the general population of clinicians. Other limitations relate to the necessary trial modifications which have been made to ensure its feasibility. This includes a shift from an original cluster RCT design to a single-arm study design, following community consultation processes. However, the revised design will ensure that individual supports can be provided by the research team to each participant, including those who have experienced a history of sexual trauma, and avoid distress associated with randomisation to a control group.

In conclusion, the world now has the tools and the technology to eliminate cervical cancer as a public health problem within the next one hundred years, and Australia is on track to reach this target by as early as 2035.^1^ However, no one must be left behind, including people with intellectual disability. It is essential that funding efforts are directed to ensuring equity across the three pillars of elimination including access to HPV vaccination, to cervical screening and to treatment of precancer and cancer, and that national data collection through digital registries is available to track progress against the global targets, including for under-screened groups. The co-produced ScreenEQUAL accessible multimodal information resource, supporting materials and healthcare provider trauma-informed training materials have the potential for national scale-up, and to be adapted to other country contexts including those in in resource-constrained settings. The study outputs will, it is hoped, help ensure elimination of cervical cancer is achieved equitably.

## Ethics statement

All members of the investigative team agree to adhere to the National Health and Medical Research Council of Australia (NHMRC) Guidelines for the Responsible Conduct of Practice according to the Declaration of Helsinki, and section 4.5.10 of the Australian National Statement on Ethical Conduct in Human Research for obtaining informed consent for people with cognitive impairment or an intellectual disability. Stage 1 of our study was approved by the University of Sydney Human Research Ethics Committee (HREC project number: 2023/146). The Stage 2 main trial and sub-studies will be registered with the Australian and New Zealand Clinical Trials Registry (ANZCTR) and ClinicalTrials.gov following full ethics approval from the University of Sydney Research Ethics Office. Any significant ongoing protocol changes will be submitted as an amendment to the University of Sydney Research Ethics Office for approval, with significant changes updated on the Australian and New Zealand Clinical Trials Registry (ANZCTR) and reflected in the final outcomes paper. Upon conclusion of the trial, results will also be reported on the ANZCTR. A final electronic de-identified qualitative and quantitative data set produced by this research will be stored in a searchable repository, available on request, following approval from the University of Sydney Research Ethics Office, for retrieval and analysis.

## Author contributions

DB: Conceptualization, Funding acquisition, Investigation, Methodology, Writing – original draft, Writing – review & editing, Formal analysis. JU: Conceptualization, Funding acquisition, Investigation, Methodology, Writing – review & editing, Formal analysis. IS: Conceptualization, Funding acquisition, Investigation, Methodology, Writing – review & editing, Formal analysis. JL: Conceptualization, Formal analysis, Funding acquisition, Investigation, Methodology, Writing – review & editing. MD: Funding acquisition, Investigation, Methodology, Writing – review & editing, Data curation, Formal Analysis. E-LC: Conceptualization, Funding acquisition, Investigation, Methodology, Writing – review & editing, Formal analysis. AC: Conceptualization, Funding acquisition, Investigation, Methodology, Writing – review & editing, Formal analysis. SS: Funding acquisition, Investigation, Methodology, Writing – review & editing, Formal analysis. LW: Project administration, Writing – review & editing, Formal analysis. RP: Project administration, Writing – review & editing, Formal analysis. CB: Project administration, Writing – review & editing, Formal analysis. EK: Project administration, Writing – review & editing, Formal analysis. HJ: Project administration, Writing – review & editing, Formal analysis.

## Footnotes

1 https://miro.com/.

2 https://pubmed.ncbi.nlm.nih.gov/30291040/.
