# Supplementary material for: Working together with people with intellectual disability to make a difference: a protocol for a mixed-method co-production study to address inequities in cervical screening participation
Source: Front Public Health. 2024 May 23;12:1360447. doi: 10.3389/fpubh.2024.1360447 (PMC11155193; doi:10.3389/fpubh.2024.1360447)
Supplement: Supplementary file 1 [file Presentation_1.pdf]

Easy Read Version

Working together with people with intellectual disability to improve cervical screening

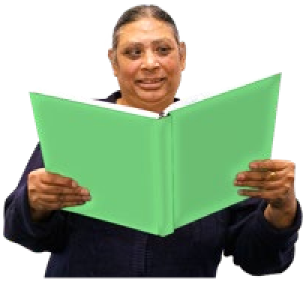

This booklet is about a research study on

- people with intellectual disability having a **cervical screening test**

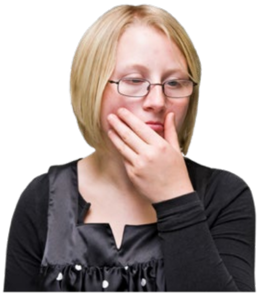

If anything in this booklet makes you sad

- please ask someone you trust for help

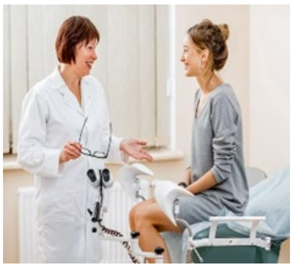

A Cervical Screening Test

- replaces the 'Pap smear'
- should be done every 5 years
- can protect you from having **cervical cancer**

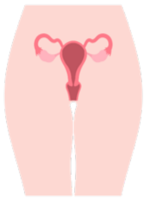

Cervical cancer is

- one of the most stoppable cancers
- caused by the **human papillomavirus infection (HPV for short)**

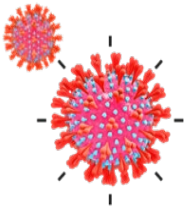

HPV can cause changes in the **cervix**

- that could lead to cancer

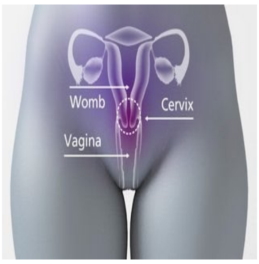

The **cervix** is inside your body

- at the top of your vagina

What we know?

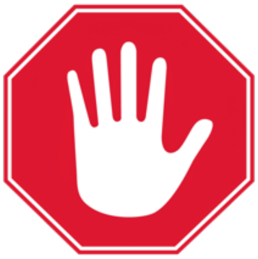

Cervical cancer can be stopped

- if all people with a cervix
- have the cervical screening test
- including people with intellectual disability

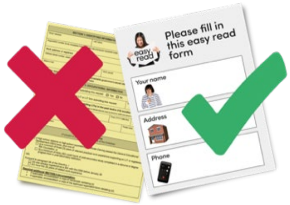

In Australia the government sends people a letter

- about the cervical screening test

It is not in Easy Read or plain English

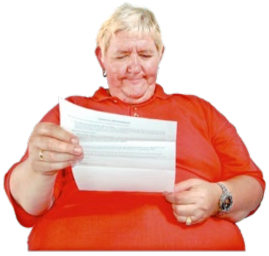

But people with intellectual disability

- cannot always read the letter or understand it
- do not always get the letter

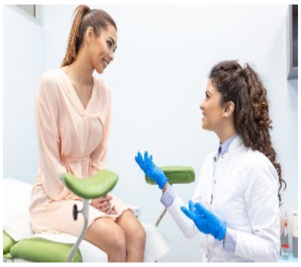

People with intellectual disability often do not get

- the cervical screening test

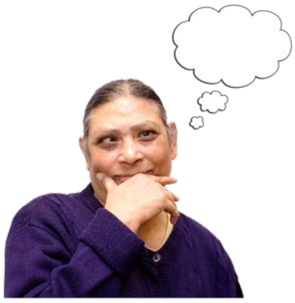

This is because they do not know it is important

Health care workers and families also may not

- think it is important for them
- know about it

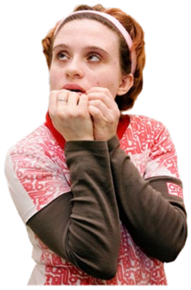

People with intellectual disability do not get cervical screening

- because they are scared

Many had bad experiences with health care workers

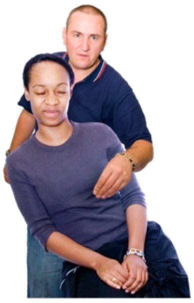

Many were also sexually abused

- cervical screening reminds them about it

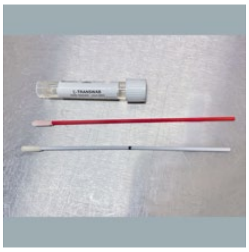

There is a new way to do cervical screening

- it is called **self-collection**

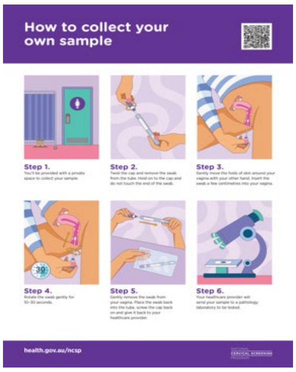

Self-collection means that

- people can test themselves with a tool

Doctors or nurses can also use the tool

- to test people if the person prefers

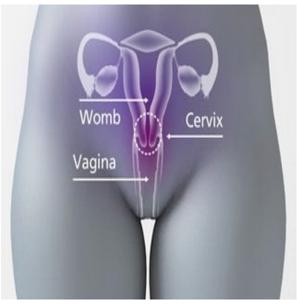

You test yourself by

- putting the tool up into your vagina
- and turning it for 20 seconds

What is the problem?

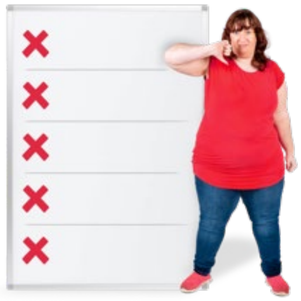

In Australia, most people who get cervical cancer

- do not get cervical screening

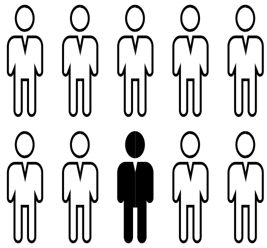

Who gets cervical screening?

- 7 out of 10 people without disability
- 1 out of 10 people with intellectual disability

How do we want to solve the problem?

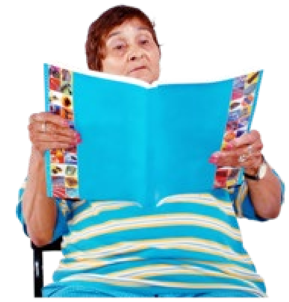

The ScreenEQUAL researchers plan to **co-produce**

- Easy Read booklets
- Videos

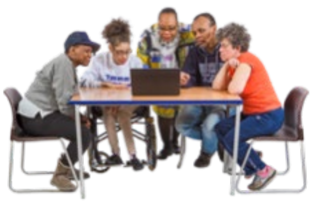

Co-produce means

- people with and without disability
- work together

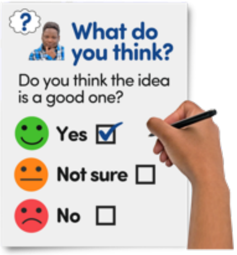

The ScreenEQUAL team will talk to people to

- find out what is helpful and unhelpful
- about having a cervical screening test

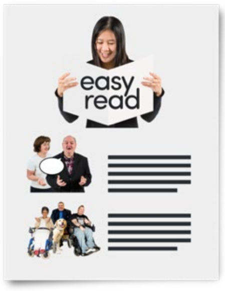

This will help to make Easy Read booklets and videos for

- people with intellectual disability
- their families
- disability support workers
- organisations
- doctors and nurse

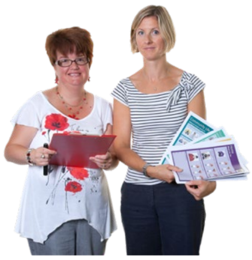

The ScreenEQUAL team will try out the resources

- to see if they are helpful

How was the study made?

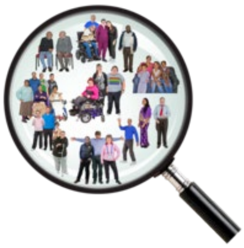

The ScreenEQUAL team is an inclusive team

People with and without intellectual disability are part of the team

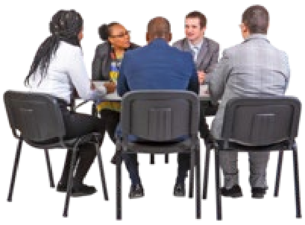

A group of people called the Advisory Group

- helps the team

They meet with the team 2 times a year

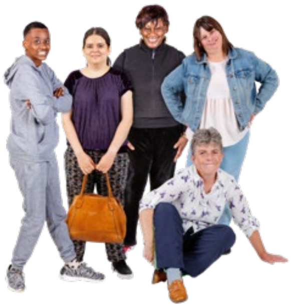

People in the Advisory Group are

- people with intellectual disability
- cancer and health care providers
- other disability groups
- Aboriginal and Torres Strait Islander people
- culturally and linguistically diverse people

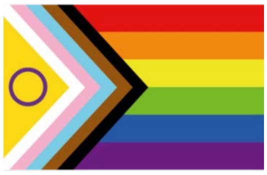

- people from Lesbian, Gay, Bisexual, Transgender, Queer, or questioning and other gender diverse identity groups (LGBTQ+)

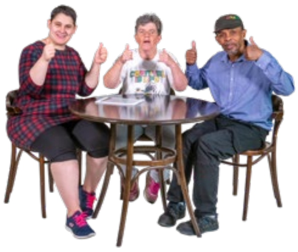

They listen to what the team does

They tell the team

- what was right and wrong
- what they should do

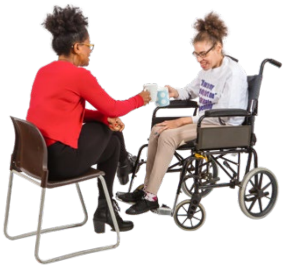

The team uses a **trauma informed approach**

This means if a person had bad experience

- they are supported in the interview

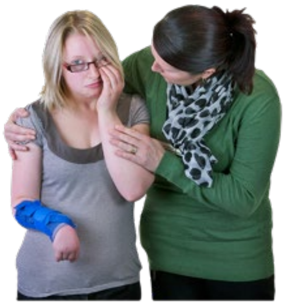

If they are upset the interview is stopped

- and people are supported

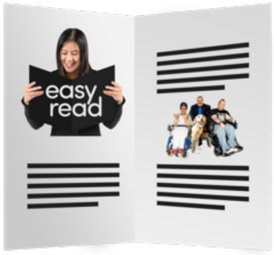

The team will write Easy Read documents about

- what we found out
- what we did about it

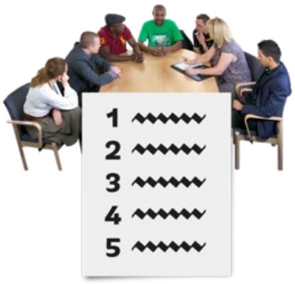

The research team uses

- pictures
- videos
- role-plays
- workshops

to help people understand the information

We will try out the resources

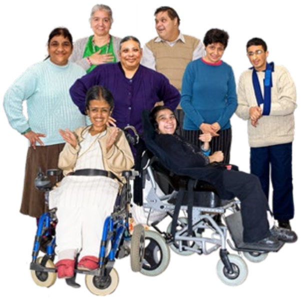

The original plan needed a lot of people

- to try out resources to check if they are helpful

But it was not possible

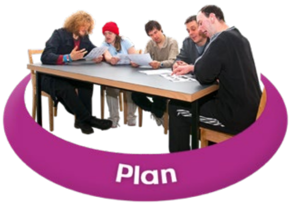

But the team made a new plan

- with less people to try out the resources

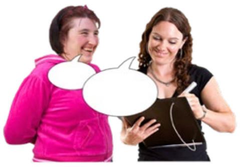

Disability support groups tell people about the study

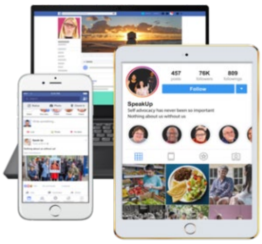

They use social media to ask people

- if they would like to be part of this study

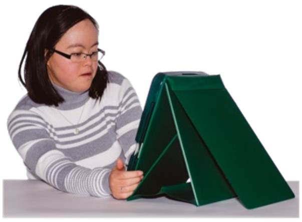

The team uses Easy Read and communication devices

- so people can understand the study

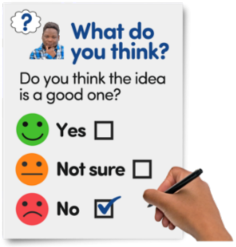

If a person changes their mind

- they can leave the study at any time

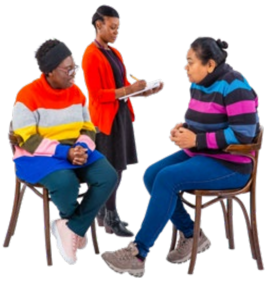

People with intellectual disability

- are from cities and rural areas
- in New South Wales, Australia

They are interviewed in person

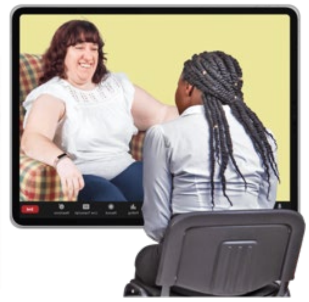

These people are from all Australian states

- family members
- support people
- doctors and nurses
- disability support agencies

They are each interviewed online

Part 1: Interviews

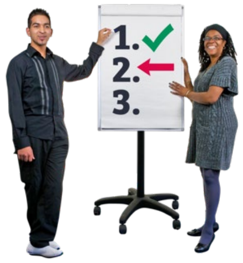

The study has 2 parts

- part 1 is interviews

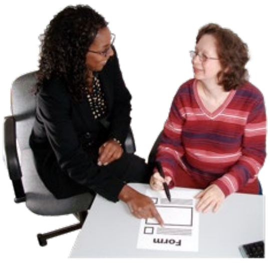

Interviews are about cervical screening

- what is helpful and unhelpful
- for people with intellectual disability

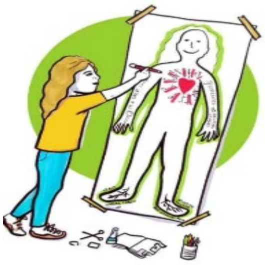

People can also do **body mapping**

- This helps people talk
- about their experiences

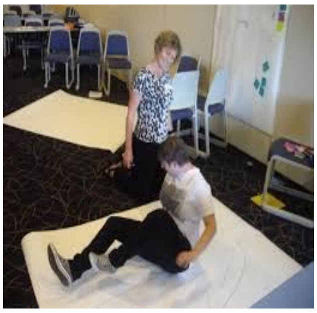

In **a body mapping** interview

- a person lies on the floor
- a researcher draws an outline of their body

Or the team has already made body outline

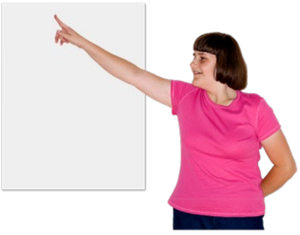

People can choose

- how they want to make their body map

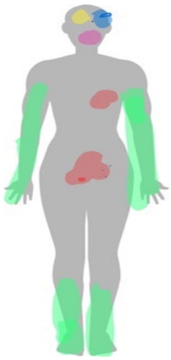

On their body map they can

- use colours and stickers
- write words and draw
- glue in pictures from magazines

to show how they felt during a cervical screening test

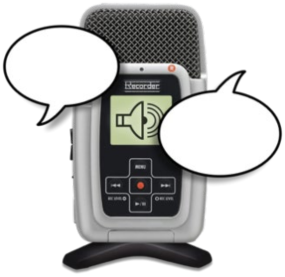

Interviews are recorded

The team uses made up names

- for people who take part

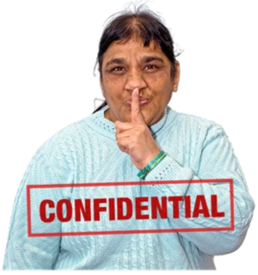

Everything is kept safely

- at Sydney University in Australia

Making resources

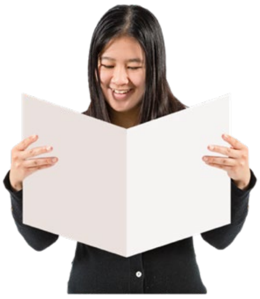

Helpful resources will be made for

- people with intellectual disability
- families and disability support workers
- organisations
- doctors and nurses

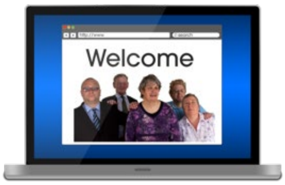

A website will be made

- to keep the new resources

Part 2: Trying out resources

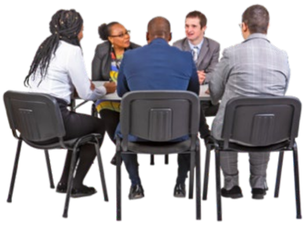

People will be asked about cervical screening

- what they think about it
- will they choose to be screened

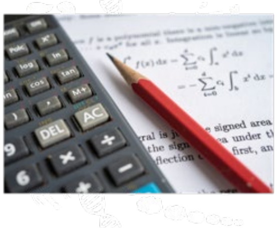

The findings will tell ScreenEQUAL team

- if the resources are helpful

To sum up

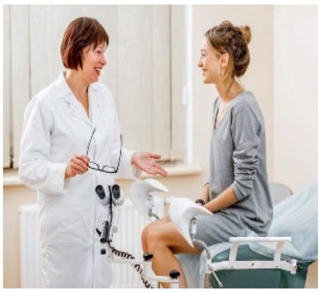

People with intellectual disability

- are not always screened for cervical cancer

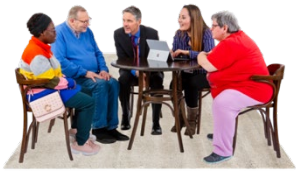

Resources will be made with people with disability to

- help people get screened to stop cervical cancer

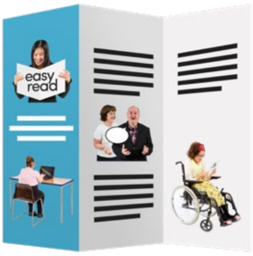

The ScreenEQUAL team made this booklet

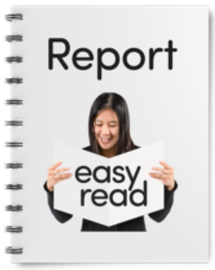

This booklet is an Easy Read report about

- cervical screening for people with intellectual disability

It was made with Photosymbols and Canva

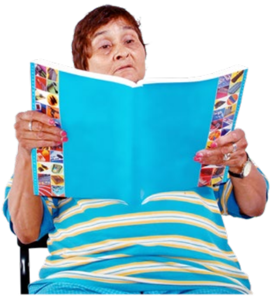

The full article is called:

“Working together with people with intellectual disability to make a difference: A protocol for a mixed-method co-production study to address inequities in cervical screening participation”

Scan the QR code for the full article

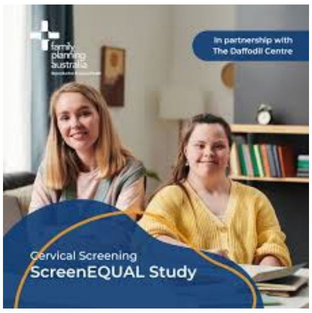

The ScreenEQUAL website has more information

https://daffodilcentre.org/news/screenequal-increasing-equitable-access-to-cervical-screening-in-australia/
